# Impact of Aging on Empathy: Review of Psychological and Neural Mechanisms

**DOI:** 10.3389/fpsyt.2019.00331

**Published:** 2019-06-11

**Authors:** Janelle N. Beadle, Christine E. de la Vega

**Affiliations:** Department of Gerontology, University of Nebraska at Omaha, Omaha, NE, United States

**Keywords:** empathy, aging, neuroimaging, theory of mind, prosocial behavior

## Abstract

Empathy in aging is a key capacity because it affects the quality of older adults’ relationships and reduced levels are associated with greater loneliness. Many older adults also find themselves in the role of a caregiver to a loved one, and thus empathy is critical for the success of the caregiver–patient relationship. Furthermore, older adults are motivated to make strong emotional connections with others, as highlighted in the socioemotional selectivity theory. Consequently, reductions in empathy could negatively impact their goals. However, there is growing evidence that older adults experience at least some changes in empathy, depending on the domain. Specifically, the state of the research is that older adults have lower cognitive empathy (i.e., the ability to understand others’ thoughts and feelings) than younger adults, but similar and in some cases even higher levels of emotional empathy (i.e., the ability to feel emotions that are similar to others’ or feel compassion for them). A small number of studies have examined the neural mechanisms for age-related differences in empathy and have found reduced activity in a key brain area associated with cognitive empathy. However, more research is needed to further characterize how brain changes impact empathy with age, especially in the emotional domain of empathy. In this review, we discuss the current state of the research on age-related differences in the psychological and neural bases of empathy, with a specific comparison of the cognitive versus emotional components. Finally, we highlight new directions for research in this area and examine the implications of age-related differences in empathy for older adults.

## Defining Empathy

Empathy is thought to be made up of two primary components, which include 1) emotional empathy—the capacity to feel either compassion or similar emotions to what another person is experiencing—and 2) cognitive empathy—the capacity to take the mental perspective of others and understand their thoughts and feelings (1, 2). Within each component of empathy, there are subdomains that are purported to have different influences on emotion, well-being, and prosocial behaviors towards others, such as donation and volunteerism (1, 3–5). Empathy can be measured as a general tendency (i.e., trait), a momentary emotional response (i.e., state), or as a behavioral response (1, 2, 4).

Empathy tends to occur when we observe others’ physical and/or emotional suffering (1). Emotional empathy is the affective reaction to others’ pain that entails feeling emotions that are the same or similar to that of the person who is suffering, feeling sympathy, or experiencing feelings of distress (4). An individual may experience compassion or sympathy in response to another person’s suffering, which is a subdomain of emotional empathy called “empathic concern” (1, 2). This is thought to be the result of engaging one’s emotion regulation capacities in order to reduce the levels of negative emotions one might experience when viewing another’s pain. In contrast, if an individual is not able to regulate their negative vicarious emotions due to observing others’ pain, they may experience the subdomain of empathic, or personal distress, which is a feeling of being overwhelmed with anxiety, distress, and negative emotions due to experiencing others’ pain (1, 2). In addition to emotion regulation, other psychological interventions may be useful in increasing levels of empathic concern and reducing personal distress, such as engaging in loving kindness meditation (4).

The cognitive component of empathy includes one’s capacity to understand the thoughts and feelings of others who are suffering (6, 7). One subdomain of cognitive empathy is perspective taking, which involves mentally putting oneself in another person’s shoes in order to understand their thoughts and feelings. Perspective taking may engage such processes as imagination, autobiographical memory, and future thinking as an individual attempts to determine another person’s thoughts and feelings. There is also evidence that actively attempting to engage in perspective taking can result in an increase in momentary levels of emotional empathy (1). Another subdomain of cognitive empathy is theory of mind, which involves accurately detecting others’ mental states and allows us to understand that others may have different perspectives from our own. Empathic accuracy is the capacity to detect fine-grained changes in the thoughts and emotions of others that may rely on capacities such as emotion recognition and social knowledge. These subdomains may interact so that individuals can accurately detect the thoughts, emotions, and intentions of others. In the following sections, we will discuss age-related differences in the psychological and neural mechanisms of emotional and cognitive empathy.

## Importance of Empathy in Aging

Currently, adults 65 years and older make up 15% of the United States population, and by 2060, this percentage is expected to nearly double to 24% (8). Therefore, an understanding of how healthy aging affects the brain and cognitive and emotional processing is critical for older adults’ well-being. Of particular importance, there is growing evidence that older adults experience at least some adverse changes in empathy (3, 9, 10), although the nature of the effect appears to differ based on the subtype of empathy. Briefly, in the field of empathy research, an important distinction has been made between the cognitive subtype (i.e., the capacity to understand others’ feelings) and the emotional subtype (i.e., the capacity to experience similar emotions to others or to feel compassion), as these components have been shown to involve distinct neural and psychological processes (1, 11–14).

Clarifying how aging affects the biological and psychological processes underlying the subtypes of empathy is a serious public health concern, as reduced empathy in general has been associated with greater risk for loneliness and depression, and poorer personal life satisfaction (15–17), which are all major concerns that have been tied to increased morbidity in older adults (18). Furthermore, loss of emotional and/or cognitive empathy has emerged as a key symptom in patients with Alzheimer’s disease and frontotemporal dementia, and some have suggested that these measures may even help to distinguish between the two conditions (19–24). There is evidence that as individuals age, they may experience higher levels of well-being (25), and that lower well-being is closely tied with increased mortality risk. In particular, a Gallup World Poll showed that the relationship between evaluative well-being (or life satisfaction) can be described as a U-shaped curve, reflecting greater life satisfaction with age; however, there are geographical differences (25).

Changes in empathy with age could have significant ramifications for older adults involved in helping roles, such as physicians or family caregivers. Many older adults find themselves to be in the role of a family caregiver to a spouse or parent with dementia, or another chronic disease (26). Both physician and caregiver burnout and compassion fatigue are already significant and pervasive issues (27, 28), and thus empathy changes as a function of aging could critically affect these professions, in addition to reducing quality care for patients. Taken together, it is clear that developing a greater understanding of how aging affects the neural and psychological factors subserving each subtype of empathy is a critical step in moving the field forward, especially with our increasing older population.

## Aging and Empathy: Psychological Mechanisms

### Aging and Emotional Empathy

There is growing evidence that the emotional domain of empathy is not lower in older than in younger adults (3, 5, 9, 10) (see **Table 1**). However, there is mixed evidence about whether older adults have similar levels of emotional empathy to younger adults (i.e., preservation) or experience higher levels (3, 5, 10). In the next section, we will review studies examining the degree to which there are age-related differences in emotional empathy between older adults and younger adults. In particular, we will clarify whether there are age-related differences in older adults’ emotional empathy among the various subdomains, including the capacity to experience the same or similar emotions to others who are suffering (emotional resonance), feelings of compassion for others (empathic concern), and distressing feelings (personal distress). We will also compare the results of studies that have assessed emotional empathy as a general tendency (or trait) versus those that have measured it as a momentary response (state) to environmental stimuli that are likely to evoke an empathic state. Finally, we will also discuss the effects of demographic factors (e.g., gender, culture) and contextual factors (e.g., age relevance of stimuli).

**Table 1:** Emotional empathy and aging: Review of findings.

Authors	Age group	Measurement	Difference	Findings
Bailey et al. (9)	YA, OA	EQ emotional empathy	OA vs YA: n.d.	OA vs YA: n.d. on self-report emotional empathy
Bailey et al. (29)	YA, OA	IRI PD, EC, ERS	OA vs YA: n.d.	OA vs YA: n.d. on emotional empathy; state PD: OA > YA; helping effort: OA > YA
Beadle et al. (10)	YA, OA	IRI EC	OA vs YA: n.d.	IRI-OA vs YA: n.d. on self-report empathic concern
Beadle et al. (3)	YA, OA	IRI EC, ERS, DG	OA vs YA IRI EC: n.d., OA vs YA ERS: n.d., OA > YA DG – empathy condition	OA vs YA self report empathic concern: n.d., OA vs YA state empathy: n.d., OA > YA prosocial behavior – empathy condition
Chen et al. (30)	YA, MA, OA	IRI, fMRI	OA < YA IRI EC and PD; OA < MA/YA brain activity in right insula to empathy condition	IRI-OA ↓ self-report on emotional empathy (EC and PD); OA ↓ brain activity in right insula to empathy for physical pain
Khanjani et al. (31)	AD, YA, MA, OA	EQ emotional empathy	OA > AD	OA > AD on EQ emotional empathy
Sze et al. (5)	YA, MA, OA	ERS, donation	OA > MA > YA ERS OA > MA > YA donation	ERS-OA ↑ than MA and YA on state empathy, OA ↑ on donation behavior
Moore et al. (32)	OA	MET, AFM, GNG, N-back, fMRI	OA with higher emotional empathy < bilateral amygdala and R insula during N-back	OA with higher emotional empathy outside scanner < bilateral amygdala and R insula during N-back task
Riva et al. (33)	AD, YA, OA		OA < YA R insula in empathy conditions	OA < YA R insula in pleasant and unpleasant touch empathy conditions

Age groups: AD, adolescents; YA, young adults; MA, middle age adults, OA, older adults; EQ, Empathy Quotient; IRI, Interpersonal Reactivity Index; EC, empathic concern; PD, personal distress; MRI, magnetic resonance imaging; fMRI, functional magnetic resonance imaging; ERS, emotional response scale (measures state emotional empathy); DG, dictator game (measure of prosocial behavior); MET, Multifaceted Empathy Test; AFM, Affective Facial Matching Test; GNG, Go/no-go Test; N-back, N-back test; R, right side; n.d., groups were not statistically different.

#### Emotional Empathy—Trait

Trait emotional empathy can be measured through self-report questionnaires in which an individual is asked to rate the degree to which they agree with a series of statements reflecting actions and thoughts about individuals who are suffering or in need (2). One of the mostly widely used assessments of trait emotional empathy in aging is the well-validated and reliable Interpersonal Reactivity Index (IRI) (2), which has sufficient test/retest reliability (range: *r* = .61 to .81) and internal consistency (range Cronbach’s alpha: .68 to .79). Although the IRI measures multiple components of empathy, here we focus on the subscales most relevant to emotional empathy, which include the Empathic Concern subscale, and the Personal Distress subscale. Each subscale ranges from 0 to 28 points, with higher scores indicating greater empathy. Another frequently used measure of trait empathy is the Empathy Quotient, which is a self-report measure that assesses cognitive and emotional empathy, in addition to social skills (34). Most relevant to this section is the affective empathy subscale. An example item from this subscale is, “Seeing people cry doesn’t really upset me,” which would be reversed scored. Higher scores on this measure indicate greater affective empathy.

Overall, most studies have not found lower trait emotional empathy in older adults than younger adults, and the majority of studies have found no age-related differences. Across two studies, Bailey and colleagues found no age-related differences in trait emotional empathy, which included two samples of participants from Australia (9, 29). In the first study by Bailey and colleagues (35), trait emotional empathy was measured by the affective empathy subscale of the Empathy Quotient and assessed age-related differences between younger (*N* = 80; 19–25 years; 29% male) and older adults (*N* = 49; 65–87 years; 33% male). They found no significant age-related differences on this measure (9). The second study by Bailey and colleagues used the IRI to measure age-related differences between younger (*N* = 40; 17–29 years; 30% male) and older adults (*N* = 39; 61–82 years; 36% male) in trait empathic concern and personal distress (29); no age-related differences in trait emotional empathy were found. Consistent with the Bailey and colleagues studies, our group found no age-related differences in trait empathic concern across two different studies with participant samples from the United States (3, 10). We used the IRI Empathic Concern subscale to assess empathic concern, and in both samples approximately 60% were women (3, 10).

A study by Chen and colleagues examined trait empathic concern and personal distress in a sample of ethnic Chinese participants including three groups: younger (*N* = 22; 20–35 years; 50% male), middle-aged (*N* = 22; 40–55 years; 50% male), and older participants (*N* = 21; 65–80 years; 52% male). In contrast to the other studies, Chen and colleagues found that older adults reported lower trait empathic concern and personal distress than the younger group (30). There are several possibilites as to why the Chen and colleagues study is not consistent with the other studies. For instance, the Chen and colleagues study included a sample of ethnic Chinese participants, whereas the other studies on this topic had samples based in Australia or the United States. Thus, there is a possibility that cultural differences in perceptions about empathy may interact with age-related differences. Another possibility is that differences in sample size across the studies could have impacted the results. The Chen and colleagues study included a smaller sample in each group (∼22 participants) than most previous studies. Consequently, this smaller sample size may have impacted the results. More research is needed to clarify the effects of these methodological differences on the measurement of age-related differences in empathy.

In summary, the studies on trait emotional empathy in general show no age-related differences between younger and older adults, with the exception of the Chen and colleagues study. Most studies focus on trait empathic concern using the IRI and show no age-related differences. However, one study also reported the results of the Personal Distress subscale of the IRI and found no age-related differences (29). Typically, sample sizes range from 40 to 80 per age group. In order to further characterize age-related differences in trait emotional empathy, more consistency is needed in terms of the sample size per age group, equivalent numbers of males and females, questionnaire type, and reporting of both empathic concern and personal distress. Furthermore, future research is needed to examine the role of culture for age-related differences in trait emotional empathy, as there is a paucity of research in this area.

#### Emotional Empathy—State

Emotional empathy can also be assessed as a state, or a momentary emotional reaction to observing the suffering of others (1). Experimentally, state emotional empathy is measured in response to an empathy induction designed to elicit a temporary state of emotional empathy. Two subdomains of emotional empathy are typically measured, which include empathic concern, often measured through items, such as “compassion and sympathy,” and personal distress, assessed through items, such as “distressed or upset.” Some studies also assess emotional empathy at baseline prior to the induction because of significant individual variability in emotional empathy reported at baseline. By assessing baseline emotional empathy, researchers can examine the specificity of the emotional empathy response to the empathy induction. It is also common practice to compare the results of the empathy induction to some form of control condition, for example, a video of an individual engaging in neutral, unemotional activities, in order to account for social context.

Overall, researchers have found that older adults do not have lower state emotional empathy than younger adults (3, 5, 29, 36). However, the evidence is mixed in terms of whether older adults experience similar or higher state emotional empathy than younger adults in response to empathy inductions (3, 5, 29, 36). To further elucidate why the results have been mixed, key methodological differences will be highlighted.

In a study by Sze and colleagues (5), state emotional empathy was measured in response to a series of empathy inductions in three different age groups: 71 younger (age: *M* = 23.07), 72 middle-aged (age: *M* = 44.58), and 70 older (age: *M* = 66.43). Approximately 67% of participants were female and 33% were male, and these proportions were evenly distributed across the three groups. Emotional empathy was elicited using a series of two videos. One condition included an “Uplifting Film,” which included photos of children with autism enjoying their time participating in a surf camp, and the second condition included a “Distressing Film,” which showed depictions of the Darfur crisis through photos of children, women, and men experiencing inhumane conditions and suffering. Participants rated their state emotional empathy at baseline (at the beginning of the study), and then again immediately after they watched each video. The items used to assess empathic concern were “sympathetic, moved, and compassionate.” Participants also rated their personal distress, and some basic emotions (e.g., anger, fear, and disgust). The scale ranged from, “1 = not at all; 5 = extremely.” For their analyses of empathic concern, baseline ratings of empathic concern were used as a covariate in the model. Researchers also assessed participants’ autonomic nervous system reactivity through measures of heart rate reactivity (interbeat interval), finger pulse amplitude, pulse transmission time to the finger, pulse transmission time to the ear, systolic blood pressure, diastolic blood pressure, and skin conductance.

In response to the videos designed to induce empathy, they found that older adults reported higher state empathic concern than middle-aged or younger adults, with the distressing video evoking higher empathic concern ratings than the uplifting video. Furthermore, they found that older adults reported higher personal distress in response to the distressing video than middle-aged and younger adults. There was a linear increase as a function of age in terms of autonomic activation. In particular, heart rate reactivity in the interbeat interval increased as a function of age, with older adults showing higher levels than younger adults. They also found that older adults showed the greatest prosocial behavior in the form of donating to charities associated with each video, with the greatest donations in response to the distressing film. Using a regression model, the authors determined that empathic concern ratings to the distressing film, interbeat interval reactivity to the uplifting film, and age were significant predictors of prosocial behavior, even when controlling for trait empathic concern and past donation behavior.

Our group compared state emotional empathy in 24 younger (age: *M* = 19.8 years) and 24 older adults (age: *M* = 77.9 years), and 63% of the sample was female. The premise of this study was that participants would be playing an economic game against two real participants, each of whom were in separate testing rooms. During the course of the study, the participants played the Dictator Game against the two “real” participants, which involved deciding how to split $10 with each participant (and the participant had to accept any offer they gave them). The Dictator Game offers served as the measure of prosocial behavior in this study. Participants were told that some of the study participants would be asked to write a note about an event that occurred during their weekend. The participants were asked to pick out of a hat to determine who would be a “Receiver” (i.e., person who reads the note) or a “Sender” (i.e., person who writes the note). Unknown to the participants, this was actually rigged, such that participants were always in the role of the “Receiver.” These notes were created and piloted beforehand in the lab. One note was designed to evoke an empathic state, whereas the other note served as the control (neutral) condition and was designed to elicit an unemotional state. Specifically, the empathy note described the participant’s experience finding out that they have a serious form of skin cancer. In the neutral note, the participant discussed their mundane errands and was unemotional in content. Participants read one note before each round of the Dictator Game; the order of the emotion inductions was counterbalanced across participants.

Similar to the Sze and colleagues study (5), participants rated their empathic concern, personal distress, and other basic emotions before and after each induction. Participants responded to the prompt, “Indicate to what extent you feel this way right now, that is, at the present moment,” which was adapted from the Positive and Negative Affect Schedule (PANAS) questionnaire (37). State emotional empathy was measured through the items (“sympathetic”; “compassionate”) and personal distress was measured through the items (“upset”; “distressed”) that were drawn from the Emotional Response Scale, a well-validated measure of state emotional empathy and personal distress (1, 38). The rating scale ranged from “1 (very slightly or not at all) to 5 (extremely).” We examined age-related differences in empathic concern ratings. For this analysis, we used a ratio score such that, “participants’ average rating after the empathy induction was divided by their average rating immediately prior to the empathy induction” (empathic concern ratio score: empathy induction/baseline score). We compared age-related differences in multiple emotions in the model: empathic concern, personal distress, sadness, hostility, and joviality.

We found no age-related differences for empathic concern, personal distress, or any other state emotion in response to the empathy induction. However, we did find that older adults showed greater prosocial behavior than younger adults in response to the empathy induction, but not the neutral induction. Specifically, older adults gave more money to their opponent who they thought had cancer than younger adults did. Furthermore, in the older group state, ratings of empathic concern were positively correlated with prosocial behavior. In summary, we found no age-related differences in state emotional empathy, but did find greater prosocial behavior in an empathic context in older versus younger adults.

A study by Bailey and colleagues examined age-related differences in emotional empathy for the physical pain of others in a sample of 40 younger and 40 older adults (29). Participants viewed video clips of arms engaged in painful versus nonpainful movements while undergoing electromyography and rating their emotions. State emotional empathy was measured in the same way as the Beadle and colleagues (3) study, by having individuals perform ratings before and after each video, which measured state empathic concern and personal distress, in addition to other basic emotions. Furthermore, they also calculated ratio scores of state emotion in a similar manner to Beadle and colleagues (3), by calculating the average ratings in each category divided by the prestimulus baseline for that category. In addition, they measured prosocial behavior by giving the participants the option to help the people in the videos by volunteering to prepare packets for mailing that would go out to the group “Pain Australia.” Helping effort was measured as the number of pamphlets participants were able to compile divided by the digit symbol substitution test score, in order to control for age-related differences in processing speed. In terms of state emotional empathy, older adults reported greater personal distress than younger adults. However, they did not find any age-related difference in state empathic concern. Other age-related differences in ratings of state emotion included older adults reporting greater happiness, hostility, and sadness. In terms of physiological responses, older adults showed greater corrugator activity than younger adults in response to the pain videos, and older adults showed greater corrugator activity to pain versus non-pain videos. The authors did not find significant age-related differences in prosocial behavior.

A study by Wieck and Kunzmann (36), compared state empathic concern in a sample of women consisting of 101 younger (age: *M* = 24 years) and 101 older (age: *M* = 69 years) participants. The rationale for the authors’ focus on women in this paper is that there is evidence for gender differences in self-reported empathy (women > men; 2), and by focusing on women, some variability in empathy would be reduced. Participants watched a neutral video and six different empathy induction videos. The neutral video depicted a woman sharing her thoughts on her way to work, whereas the empathy videos depicted a variety of different emotions (e.g., anger, sadness, or happiness). The videos included protagonists from the community reliving specific emotional experiences. They were asked to select age-relevant experiences, such as the “death of a long-term friend” for older adults and the “end of one’s first love” for younger adults. After viewing each clip, participants were asked to rate their current emotions based on a list of emotion adjectives on a scale ranging from “0 (not at all) to 6 (extremely)” (36). For the emotion adjectives, participants rated their empathic concern through the item “sympathetic,” in addition to rating other basic emotions related to happiness, sadness, and anger. Of note, they did not assess state emotions at baseline in this study, but emotions were assessed in response to the neutral video, which served as the control condition.

Across the various types of empathy inductions, older women reported greater empathic concern (or sympathy) than younger women. However, there were slight differences in the magnitude of the response, with older women showing higher ratings of sympathy for the happiness videos versus the sadness and anger. The age relevance of the videos did not significantly impact ratings of sympathy. In terms of emotion congruence, there were few age differences. Overall, participants rated higher emotion congruence for videos depicting older topics and for happiness and anger videos. For sadness films, both younger and older participants reported greater emotional congruence with those that depicted older protagonists. In the domain of anger, older women reported greater emotion congruence with videos depicting older topics. In summary, whereas older females reported greater sympathy, there were fewer differences in emotion congruence.

Overall, no studies have reported lower state emotional empathy in older adults than younger adults in response to an empathy induction. Yet, there is still little consensus on whether older adults’ state emotional empathy is higher than or similar to that of younger adults. We have reviewed two studies that have shown no age-related differences in state empathic concern in older than in younger adults (3, 29) and two that have shown higher levels in older adults (5, 36). In terms of state personal distress, two out of three studies showed increased levels of personal distress in older adults in response to empathy inductions (5, 29), whereas the third study found no difference (3). Some researchers have measured older adults’ physiological and facial mimicry responses to empathy induction and found that older adults show greater heart rate reactivity (5) and facial corrugator activity (29). In the domain of prosocial behavior, two out of three studies found greater prosocial behavior in response to empathy inductions in the older group than the younger group (3, 5, 29). However, the study that did not show age-related differences in prosocial behavior (29) measured behavior in terms of time spent helping rather than monetary donation, which was the method used in previous studies. Previous studies vary in terms of the type of empathy induction used (e.g., note versus video induction) and the content within the induction (e.g., uplifting versus distressing; emotional versus physical pain). Furthermore, only some studies include an equal number of males and females in their sample. While prosocial behavior is most often measured through monetary donation, more research is needed to assess whether age-related differences in empathy also affect other types of helping behavior. In conclusion, future research is needed to characterize the degree to which state emotional empathy is increased in older adults, whether there are gender differences, and the degree to which increased emotional empathy affects different types of helping behavior (e.g., monetary donation versus spending time helping others).

#### Conclusions about Aging and Emotional Empathy

Across studies, there is little evidence that emotional empathy is lower in older than younger adults. However, it is still not clear whether emotional empathy is similar to or higher than in younger adults, in particular in the empathic concern domain. There is growing evidence that in the personal distress domain, older adults may experience higher levels than in younger adults. Yet, this area is still in its infancy and more research is needed to assess all subdomains of emotional empathy, including empathic concern, personal distress, and emotional resonance through both trait and state measures. For future studies that measure age-related differences in trait empathy, more consistency is needed in the questionnaires used to measure empathy, as different questionnaires may assess empathy differently. In studies that focus on state emotional empathy, in order to reach a consensus, more research is needed that characterizes empathy through the use of multiple different types of induction techniques (e.g., video, photos, and notes) and that uses both negative and positive content, as well as emotional and physical pain. In order to assess the relationship between age-related differences in empathy and prosocial behavior, more research is needed to measure different types of prosocial behavior (e.g., donation, and volunteerism). Overall, there is a need to assess the role of gender and culture for age-related differences in empathy and prosocial behavior.

Much of the research on age-related differences in emotional empathy has focused on self-report measures. Yet, self-report measures of empathy are not without their limitations. For instance, these measures may be influenced by demand characteristics, as individuals may want to appear empathetic because it is thought to be a desirable trait. Furthermore, socioemotional selectivity theory suggests that older adults may be motivated by activities that enhance emotional meaning, such as spending time with close friends and family (39). Thus, for older adults, perceiving themselves to be compassionate may be particularly relevant to their personal goals. Studies that use more objective physiological measures provide some support for increased emotional empathy in aging. In particular, older adults show greater heart rate reactivity (5) and facial corrugator activity (29) in response to empathy inductions, which is consistent with higher levels of personal distress ratings. Moving forward, it is important that studies use converging methods to assess age-related differences in empathy, such as self-report, behavioral, physiological, and neuroimaging measures.

### Aging and Cognitive Empathy

The cognitive component of empathy is thought to be made up of multiple subdomains that include theory of mind, empathic accuracy, and perspective taking (6, 7). Theory of mind is our capacity to detect the mental states and intentions of others (40–42), whereas perspective taking is the capacity to adopt the mental states of others, typically through imagining their point of view (30, 43, 44). A related construct, empathic accuracy, is our capacity to accurately discern the emotions, thoughts, and feelings of another person (30, 45–47). These three subdomains interact to enable individuals to understand the thoughts, feelings, and intentions of others. Below, we will describe age-related differences in cognitive empathy across the various subdomains of theory of mind, perspective taking, and empathic accuracy (⫔see **Table 2**).

**Table 2:** Cognitive empathy and aging: Review of findings.

Authors	Age group	Measurement	Difference	Findings
Bailey et al. (9)	YA, OA	EQ, RET	OA < YA	EQ-OA ↓ self-report on cognitive empathy, RET-OA ↓ on RET
Beadle et al. (10)	YA, OA	IRI	OA < YA	IRI-OA ↓self-report on cognitive empathy
Bottiroli et al. (48)	YA, OA (O-O, Y-O)	FPT, WMU	OA < YA	FPT-OA ↓ on cognitive ToM but not affective ToM; working memory updating mediated effect of age on cognitive ToM
Chen et al. (30)	YA, MA, OA	IRI, fMRI, FPS, CASI	OA vs YA/MA IRI PT: n.d.	No difference on IRI PT between OA/MA/YA
Duval et al. (49)	YA, MA, OA	RET, ToMS		ToMS - OA ↓, RET-OA ↓ on complex emotions
German & Hehman (50)	YA, OA	ToMS	OA < YA	OA with ↓ cognitive performance ↓ on ToMS with more executive functioning demands
Jarvis & Miller (51)	YA, OA	ToMS	OA < YA	OA ↓ on ToMS, episodic memory, and prospection; lowest score on cognitive ToMS
Khanjani et al. (31)	AD, YA, MA, OA	EQ, RET	OA > AD EQ cognitive empathy; OA < AD/YA/MA RET	OA > AD on self-report cognitive empathy; OA < AD/YA/MA on theory of mind task
Maylor et al. (52)	YA, OA (Y-O, O-O)	ToMS, WCST	O-O < YA/Y-O	ToMs-O-O/Y-O ↓ YA with memory load; O-O ↓ YA/Y-O without memory load
Moran et al. (53)	YA, OA	fMRI, FBT, MJT	OA < YA	OA ↓ on all tasks; OA ↓ dmPFC activity
Richter & Kunzmann (47)	YA, OA	EF	OA n.d. or < YA depending on context	OA ↓ on EA unless the task was motivationally relevant
Rosi et al. (41)	OA (Y-O, O-O)	ToMS (pre-test, training, post-test)	O-O < Y-O pre-tests	ToMS-O-O ↓ pre-test, both groups performed similar following training
Moore et al. (32)	OA	MET, AFM, GNG, N-back, fMRI	OA with higher cognitive empathy > insula during GNG task	OA with higher cognitive empathy > insula during GNG response inhibition task

Age groups: AD, adolescents; YA, young adults; MA, middle-aged adults; OA, older adults; Indicates older group split into a young–old and an old–old group; Y-O, young–old group; O-O, old–old group; EQ, Empathy Quotient; RET, Revised Eyes test; IRI, Interpersonal Reactivity Index; MRI, magnetic resonance imaging; fMRI, functional magnetic resonance imaging; FPT, Faux Pas test; PANAS, Positive and Negative Affect Schedule; CASI, cognitive abilities screening instrument; FPS, Facial Pain Scale; PT, perspective-taking; EA, empathic accuracy; GMV, gray matter volume; ToMS, theory of mind stories; FBT, false belief task; MJT, moral judgment task; EF, empathy films; WMU, Working Memory Updating Task; WCST, Wisconsin Card Sorting Task; MET, Multifaceted Empathy Test; AFM, Affective Facial Matching Test; GNG, Go/no-go Test; N-back, N-back test; R, right side; n.d., groups were not statistically different; ToM, theory of mind.

#### Theory of Mind

Theory of mind is described as a person’s capacity to understand the mental states of others and can include both cognitive and affective content (53–55). People may use relevant personal experiences in order to comprehend others’ cognitive and emotional states and predict social behavior (41). Theory of mind is important for social relationships and interactions because it can help us to connect with others in meaningful ways (42, 53, 56). Research on theory of mind has generally shown that older adults perform more poorly than younger adults (33, 48–53). One way to measure theory of mind is through the Faux Pas Test (57). In this task, participants are presented with short stories in which they answer questions about whether someone behaved inappropriately (i.e., committed a social faux pas) (57). Studies using this task have typically shown poorer performance in older than younger adults (48, 58).

Another way to assess theory of mind is through the False Belief Task, which is a widely used and well-validated task that involves mental state detection (59). In this task, participants read stories during two different conditions: 1) mental trials—stories about an individual who has a mistaken (or false) belief, and 2) physical trials—a physical image that is no longer accurate. The first condition will require theory of mind in order to infer the character’s mental state, whereas the physical trial will serve as the control condition. Studies using this task have shown poorer performance in older than younger adults (53, 60).

Age-related differences in theory of mind have also been assessed through the Revised Reading the Mind in the Eyes Test (54). In this task, participants are shown a series of 36 pictures, including the eye region of the face only, and each photo depicts a different social emotion. Participants determine the emotion expressed in each photo by selecting from four different emotion response options, such as “jealous” or “embarrassed” (54). In general, studies have found that older adults perform more poorly than younger adults on this task (9, 31, 61). There is some evidence that individuals with better performance on the Reading the Mind in the Eyes task have higher levels of verbal intelligence and comprehension (62). Furthermore, individual differences in verbal comprehension have been shown to mediate performance on the Eyes Task (63), and this could potentially interact with age-related differences.

In summary, across multiple different theory of mind tasks, older adults typically show poorer performance than younger adults. While there is some evidence that their decreased performance may be due to declines in certain cognitive domains (e.g., working memory or executive function), studies have shown that theory of mind is distinct from other measures of cognition. More research is needed to clarify the exact contribution of age-related changes in cognition to theory of mind performance in aging.

#### Perspective Taking

In general, older adults report lower perspective taking than younger adults, but there is still a lack of consensus in this area (3, 30, 31). Perspective taking is typically measured through the IRI (2) or the EQ (32), which are two self-report questionnaire measures of trait empathy that we have discussed in the sections on emotional empathy. The IRI has a perspective taking subscale and the EQ has a cognitive empathy subscale that measure an individual’s general tendency to adopt the thoughts and feelings of others, in order to understand their emotions.

Four studies using the IRI, EQ, or other similar measures of trait empathy have shown that older adults report lower cognitive empathy than younger adults (3, 9, 10, 64), whereas one study found no difference (30), and another study showed higher levels in older adults (65). A task-based study measuring cognitive empathy through perspective taking in the context of stories included 20 younger adults (18–29 years) and 20 older adults (64–88 years; 66). The authors found that the older adult group used more positive words for perspective-taking than the younger adult group (66). In summary, there is growing evidence that older adults have lower cognitive empathy than younger adults, but not all studies show consistent results. Thus, more research is needed to assess cognitive empathy using both self-report and task-based measures. Furthermore, there is a need to conduct longitudinal studies examining cognitive empathy to disentangle generational from age-related effects.

#### Empathic Accuracy

Several studies have examined age-related differences in empathic accuracy (30, 46, 47, 67). One study utilized the FACES task (68) which included photos depicting facial expressions from which participants (younger and older couples) were asked to identify the emotion in the photo (46). On the FACES task, older adults performed more poorly than younger adults. They were also asked to identify the emotions of their partner (in addition to their own emotions) (46) and were asked to indicate whether their partner was in view (visible to them) when they were making their emotional judgments. The visibility of their partner did not impact the older adults’ results, but for younger adults, they showed greater accuracy in predicting their partners’ emotions when they were in view of them (46).

In another study of age-related differences in empathic accuracy, older adults showed greater performance on tasks that were relevant to them (47). This finding is supported by the socioemotional selectivity theory (39), which suggests that older adults prioritize situations that are emotionally meaningful to them. The authors concluded that the older adults’ empathic accuracy performance in this situation may have less to do with their emotion recognition capacities, but instead relate to their prioritization of emotionally meaningful situations (47). In sum, this research suggests that, in general, older adults perform more poorly than younger adults on tests of empathic accuracy, except in cases where the information is emotionally relevant to them.

#### Conclusions about Aging and Cognitive Empathy

Overall, older adults tend to show reduced performance and report lower levels of cognitive empathy. In the theory of mind domain, older adults consistently show lower performance on most tasks assessing theory of mind across verbal and nonverbal measures. In terms of self-reported cognitive empathy, most studies show that older adults report lower levels than younger adults, but there a few exceptions. In the domain of empathic accuracy, there is evidence that older adults perform more poorly, except in cases where the information is highly age-relevant. In summary, the current consensus is that older adults have lower cognitive empathy than younger adults. However, future research is needed to clarify the role of other cognitive factors such as memory, attention, executive function, and verbal comprehension in age-related differences in cognitive empathy. Finally, more research is needed to elucidate the degree to which age-related differences in cognitive empathy can be consistently measured in the same individuals through behavioral versus self-report measures.

## Aging and Empathy: Neural Mechanisms

### Overview of Brain Networks Involved in Empathy

The emotional and cognitive components of empathy are thought to recruit largely distinct brain networks in younger adults (11–14). Studies examining the neural bases of empathy have used methods such as neuroimaging and lesion studies of patients with brain damage (13, 53, 69, 70). Key brain regions thought to be involved in emotional empathy include the anterior cingulate and insula, ventromedial prefrontal cortex, and amygdala (13, 69, 70). In contrast, cognitive empathy involves neural systems supporting self-projection into another person’s mind, future thinking, and episodic memory. The key brain structures involved in cognitive empathy are thought to include medial prefrontal cortex, temporal pole, posterior superior temporal sulcus, and hippocampus (53).

### Aging and Neural Correlates of Emotional Empathy

Little is known about the neural mechanisms supporting emotional empathy in aging. Three studies have investigated this question using fMRI (30, 32, 33). Moore and colleagues (33) conducted an exploratory study on the neural bases of empathy in 30 older adults (age: *M* = 79 years), but did not include a younger group comparison. For the purposes of this section, we focus on their investigation of emotional empathy, but they also assessed cognitive empathy in this study. In order to compare individuals with very high and very low empathy, they recruited from a previous registry based on empathy scores in the top decile and bottom decile of a self-report empathy questionnaire. Participants completed three tasks measuring cognitive and emotional processing including 1) affective facial matching task, 2) the Go/No-go task, and 3) the N-back Working Memory Task.

The empathy task was completed outside of the scanner and included the Multifaceted Empathy Test (71). In this empathy task, participants viewed photographs of people containing emotional content. Emotional empathy in response to these photos was measured by having participants rate their emotional response to each of the photos. Specifically, they responded to two questions: “How calm/aroused does this picture make you feel?” and “How concerned are you for this person?” and completed a rating scale that included “1 = calm/no concern to 9 = highly aroused/highly concerned.” The first prompt was thought to assess arousal level, whereas the second prompt was thought to measure empathic concern. For the purposes of analysis, the authors chose to combine the arousal variable and the empathic concern variable in order to form a composite variable thought to be representative of emotional empathy (average of the *z*-scores).

The researchers had an *a priori* hypothesis that there would be involvement of the amygdala and insula for empathy. Thus, they used a region of interest (ROI) analysis for the bilateral amygdala and insula. The authors found that older adults with higher levels of emotional empathy outside the scanner on the Multifaceted Empathy Test showed greater deactivation in both the bilateral amygdala and the right insula while completing the N-back task (the measure of working memory). This suggests that older adults with higher emotional empathy in response to others may show decreased activation in regions relevant to emotional empathy (amygdala and insula) when experiencing greater working memory load. The authors suggest that, in this case, the amygdala may be playing a role in working memory and cognitive control and that individuals who experience high empathy may potentially experience more efficient emotion regulation in the same brain regions as those that are important for working memory.

There are some key methodological differences between the Moore and colleagues study (32) and other studies examining the neural bases of emotional empathy in aging. Specifically, because the empathy task was not conducted in the scanner, direct conclusions about the relationship between neural activations and empathic processes cannot be made. Furthermore, this study only includes an older group, and thus conclusions about age-related differences cannot be determined. In addition, this study employs a region of interest approach, and therefore the specificity of these brain regions for empathy cannot be determined. Finally, the completion of different cognitive and emotional tasks in the scanner adds further complexity to the interpretation of the findings. In this context, the finding is that older adults with higher emotional empathy on the task outside the scanner show decreased activation in empathy areas when experiencing greater working memory load. This would suggest that during a cognitive task targeted to engage brain networks associated with working memory, individuals who have higher empathy may be less likely to recruit brain regions typically implicated in emotion and empathy, such as the amygdala and insula. However, because the empathy task did not occur in the scanner, we cannot make specific conclusions about the role of these regions for empathy or comment on age-related differences.

In another study by Riva and colleagues, the researchers investigated female participants of three different age groups. The age groups included 28 adolescents (age: *M* = 15.7), 32 young adults (age: *M* = 24.5), and 28 older adults (age: *M* = 63). The study focused on the neural bases of age-related differences for empathy for physical touch. There were two main conditions in the fMRI task: 1) self: individual experienced stroking by an object that felt either unpleasant, pleasant, or neutral; and 2) other: this process occurred to another individual (actually a confederate), rather than to them. They were instructed to “empathize with the other participant by vividly imagining her feelings” (33). After each condition, the participants rated their own feelings (self condition) and that of the other person (other condition) on a scale ranging from “−10 (very unpleasant) to +10 (very pleasant).” They also assessed theory of mind using the Frith-Happé animation task (72).

For the fMRI results, they found differences in the empathy condition between older and younger adults. They conducted a categorical analysis using ANOVAs that compared the three groups and the brain activity in response to unpleasant touch versus neutral (self), unpleasant touch versus neutral (other), pleasant touch versus neutral (self), and pleasant touch versus neutral (other). Specifically, they found that older adults showed lower activity in the right anterior insula (AI) than younger adults for the empathy for unpleasant and pleasant touch. The researchers then investigated the degree to which performance on the theory of mind task explained variance in the neuroimaging results. To assess this, they included theory of mind performance as a covariate in their ANOVA analyses and found that the results did not significantly change. It could be that theory of mind is not required for processing less complex levels of emotional empathy related to touch, but may be required for more complex interpretations of others’ emotional pain as found using empathy inductions that involve stories.

Only one neuroimaging study has used a standard empathy neuroimaging task to compare older to younger adults and has included both males and females (30). This study included three participant groups: 1) 22 younger (age: *M* = 23.4), 2) 22 middle-aged (age: *M* = 43.7), and 3) 21 older adults (age: *M* = 69.4). Participants were asked to passively view images of hands and feet either in pain or not in pain, but the rest of the body, including the face, was not shown). There were four conditions: 1) Solo pain (SP): one person is present in the image and the pain is caused by accident (e.g., slamming one’s finger in a car door), 2) Solo no pain (SN): one person is present in a situation that does not cause pain (e.g., opening a door); 3) Dyad pain (DP): one person is in pain that was caused by another person (e.g., one person slamming the door on another person’s finger); 4) Dyad no pain (DN): two individuals interact but neither individual experiences pain (e.g., opening a door near another person’s arm). Emotional empathy was computed by comparing “[(SP + DP) − (SN + DN)].” This task is a well-established measure to assess emotional empathy for pain, and the stimuli have been used in previous studies (73–75).

Consistent with the Riva and colleagues study (33), Chen and colleagues found that older adults showed less activity than younger adults in the right AI in response to empathizing with others’ physical pain and that greater averseness to others’ pain was positively associated with activity in the anterior mid-cingulate cortex. These findings are consistent with two brain regions that have been implicated in the experience of empathy towards the pain of others in studies of younger adults, the AI and anterior cingulate (12, 76–79).

Overall, these studies have shown that in response to emotional empathy, older adults show reduced activity in regions typically associated with emotional empathy in younger adults (e.g., anterior cingulate and insula), despite behavioral reports of either intact or higher emotional empathy in older adults. More research is needed to characterize the mechanisms by which intact or greater emotional empathy in older than younger adults is linked to reduced activity in key emotional empathy regions. Furthermore, the methods used to assess the neural bases of emotional empathy in aging vary widely between the three studies. For instance, one study focused on older adults (with no younger group comparison), and one study included only females. Recommendations for future research include assessment of the neural bases of emotional empathy in aging through multiple, standard tests of emotional empathy, a larger sample of both women and men, and a comparison to a younger sample.

### Aging and Neural Correlates of Cognitive Empathy

There are a growing number of studies on the neural bases of age-related differences in cognitive empathy, and in general, these studies find reduced brain activity in older adults versus younger adults in key regions implicated in cognitive empathy processes in younger adults (53); however, there are some exceptions. A study by Moran and colleagues examined age-related neural differences in cognitive empathy across three tasks in younger and older adults (53). These tasks fall under the theory of mind subdomain of cognitive empathy and included an “animate movement” task, a “moral judgment” task, and a “false belief task” (53). In the animate movement task, individuals were asked to infer mental states by viewing shapes interacting in either a social or nonsocial manner (80). In the second task (the moral judgment task), participants inferred the thoughts and feelings of others in stories where the target individual behaved in ways that could be considered moral or immoral (81). Finally, the third task was the false belief task (59) that we described in the psychological mechanisms of cognitive empathy section. Briefly, in the false belief task, participants read stories about others who have a false mental belief (false belief condition) or read about a physical image that is no longer accurate (physical condition). The primary findings of this study were that across the three tasks, older adults had reduced brain activity compared to younger adults in the dorsomedial prefrontal cortex (dmPFC), which is thought to be a key region in the cognitive empathy network in younger adults (53).

Moore and colleagues (32) also measured the neural bases of cognitive empathy in aging. (This study was described in detail in the neural bases of aging-related changes in the emotional empathy section). As a reminder, participants completed a response inhibition task (Go/No-go task) while undergoing fMRI, in addition to other tasks measuring cognitive and emotional function. Cognitive empathy was assessed outside the scanner using the Multifaceted Empathy Test (71), and measures were correlated with brain activity in response to the cognitive/emotional tasks conducted in the scanner. Specifically, cognitive empathy was measured by having participants make mental state judgements about each individual they viewed in the photos. They chose between four different emotions for each photo and they were provided with feedback as to the accuracy of their response immediately after they selected their response. The authors found a relationship between brain activity in response to the Go/No-go task and levels of cognitive empathy measured outside the scanner. Specifically, older adults with higher cognitive empathy (as measured by the Multifaceted Empathy Test) had greater insula activation than older adults with lower cognitive empathy. The findings from this study suggest that within the older adult group, there may be variation in levels of cognitive empathy and those with higher cognitive empathy may show greater activity in brain regions associated with empathy. However, further research may investigate whether a similar response will be found if the empathy task is conducted inside the scanner.

In summary, a small number of studies have investigated age-related differences in the neural bases of cognitive empathy (32, 53). The study by Moran and colleagues demonstrated reduced brain activity in the dorsomedial prefrontal cortex in older than in younger adults across three tasks measuring theory of mind. The Moore and colleagues study found that higher cognitive empathy performance outside the scanner was associated with greater activity in the insula than younger adults in response to a Go/No-go task. Because cognitive empathy also involves the domains of perspective taking and empathic accuracy, more research is needed to determine whether age-related differences in neural activity extend to these domains. Furthermore, more studies are needed to confirm these findings in larger samples and with a variety of cognitive empathy measures. Finally, additional research is needed on both task-based studies of the neural bases of empathy, in addition to studies assessing resting state functional connectivity and structural differences.

### Summary of Imaging Findings and Future Research

The study of the neural bases of empathy in aging is still in its infancy. While there are a growing number of studies on this topic, more research is needed before there is conclusive evidence for age-related neural differences in the cognitive or emotional components of empathy. For the emotional component of empathy, the small number of studies on this topic point to a decrease in brain activity in older adults in key regions typically involved in emotional empathy. This is surprising given that behaviorally older adults consistently show similar or higher levels of emotional empathy than younger adults. Furthermore, more research is needed that uses gold standard tasks designed to specifically assess empathy that have also been measured in younger adults. Brain imaging studies examining cognitive empathy point to decreased activity in the dorsomedial prefrontal cortex in older adults versus younger adults during theory of mind tasks. Yet, little is known about whether this extends to other domains of cognitive empathy, such as perspective taking or empathic accuracy. Across both cognitive and emotional empathy, studies using standard empathy tasks are needed that assess the multiple subdomains of empathy. Finally, to further elucidate both functional and structural age-related differences in empathy, more research is needed using multi-modal imaging techniques, with larger, more generalizable samples including men and women, and younger and older adults.

## Preliminary Conclusions: State of the Research on Empathy in Aging

The question of how aging impacts empathy has important implications for public health because reduced empathy has been associated with greater loneliness, depression, and poorer relationship satisfaction. Socioemotional selectivity theory (39) highlights the importance of emotional meaning for older adults, and this typically takes the form of spending time with close others. Thus, if older adults experience decreases in empathy, this could have a significant, negative impact on their well-being.

Research studies focused on age-related psychological differences in empathy suggest that older adults have lower cognitive empathy and preserved or increased emotional empathy; however, there are exceptions. There is growing evidence that older adults show increased prosocial behavior towards others in the form of monetary donation in response to an empathic context. Overall, the lack of consistent results in the behavioral literature may be due to the large variation in methods used to measure empathy, inconsistent sample sizes, unequal numbers of men and women, and reduced capacity to generalize across cultures.

Only a small number of studies have examined the neural bases of empathy in aging. Across both the cognitive and emotional components of empathy, older adults show decreases in key regions thought to be involved in empathy relative to younger adults. In the cognitive component, older adults show reduced activity in the dmPFC relative to younger adults. For emotional empathy, older adults show reduced activity in the anterior cingulate and insula. The findings for emotional empathy are counterintuitive because, behaviorally, older adults show similar or higher levels of emotional empathy to younger adults. More research is needed with standard empathy tasks to further characterize the neural bases of empathy in aging.

The mixed findings in the literature on empathy and aging may be partially explained by the large variation in behavioral and neuroimaging methods used to study empathy. There are a few key areas where more research is needed. One, greater consistency is needed across studies to employ gold standard tasks assessing cognitive and emotional empathy to allow for direct comparisons across studies. Two, there is significant variability in how the age groups are defined, and thus consistent age ranges across studies are needed. Three, studies should be powered to compare sex differences in addition to age differences, as key sex differences in empathy have been reported in studies of younger adults. Four, both structural and functional neuroimaging techniques are needed to further characterize the neural bases of empathy. Five, more research is needed to better understand how culture may impact age-related differences in empathy. Finally, more research is needed to investigate this question longitudinally, in particular in task-based studies of empathy to tease apart cohort effects versus actual age-related differences. Taken together, the study of age-related differences in empathy is a growing area of research that has important implications for older adult well-being and health.

## Author Contributions

JB contributed to the conceptualization and theoretical discussion within the paper and wrote and edited the paper. CV provided feedback on the conceptualization of the paper and wrote and edited the paper.

## Funding

JB’s research is funded by start-up funds from the University of Nebraska Program of Excellence Funds, the Vada Kinman Oldfield Award for Alzheimer’s Disease research, the National Science Foundation award (Co-Investigator; NSF #1539067), and NASA Nebraska EPSCoR Mini-Grant (Federal Award #NNX15AK50A). This project was also supported by the National Institute of General Medical Sciences, 1U54GM115458-01.

## Conflict of Interest Statement

The authors declare that the research was conducted in the absence of any commercial or financial relationships that could be construed as a potential conflict of interest.
